# Institutional Notes and News

**Published:** 1909-12-18

**Authors:** 


### Institutional Notes and News.

### WORCESTER GENERAL INFIRMARY.

The Ladies' Guild of this institution held its annual meeting in the board room. There was a good gathering of members, and the Countess Beauchamp presided. The report states that 114 garments were distributed for making during the course of the year. The statement of accounts shows an increase in the subscriptions of 18s. 6d. and a balance in hand of 19s. On the conclusion of the meeting the ladies visited the wards and saw many patients wearing garments of their providing. This society exists for the purpose of supplying clothing, such as flannel jackets, shirts, gowns, etc., for use during the patients' stay in hospital, and renders good and useful service thereby. At the monthly meeting of governors, Mr. T. Butler, junr., F.R.C.S., was elected hon. surgeon. The question of the reorganising of the laundry was again discussed, and referred to a sub-committee for the preparation of a scheme for dealing with the matter. The proposed Children's Block was referred to, and the motion that the hon. medical staff be requested to consider the subject and formulate a plan for further discussion was carried. It was recognised that to erect such a block would necessitate either selling out stock or making an appeal to the public, but confidence was expressed in the generosity of the inhabitants of the county to support any effort for the benefit of the children.

### THE INFECTIOUS DISEASES HOSPITAL, CLIFTON.

As the result of certain serious charges made by patients and relatives of patients against the Infectious Diseases Hospital at Clifton, the Brighouse Joint Hospital Board have unanimously come to the following agreement : "The Hospital Board have carefully investigated the complaint made by Mr. Samuel Bottomley, of Clifton, as to the treatment of his child Hilda whilst in hospital at Clifton, and after the examination of many witnesses they find that while some complaints fall to the ground in the want of confirmation, they, however, regret to find the following : That there has been a want of courtesy shown in the treatment of Mr. Bottomley when making inquiries, and that Hilda Bottomley was discharged too soon. As regards the dietary scales, there is a conflict of evidence between the hospital staff and the patients, and the Board will inquire further into these. The Board regret that there are good ground for believing there has been some laxity in the administration of the hospital, and they are determined that this state of things shall be remedied forthwith."

### GOOD FINANCE.

At the annual meeting of the West of England Eye Infirmary, Exeter, it was stated that in consequence of the resolution that the new wing, to be erected to commemorate the centenary of the institution, should not be commenced until the whole of the amount required was in hand, a letter had been received from a lady, who desired to remain anonymous, inquiring what balance was required to commence the work. On being informed that the amount was £334 1s. 3d., she generously sent her cheque for that sum. Almost simultaneously the munificent donation of £1,000 was received from Mrs. Nosworthy, of Dawlish. The Committee is consequently relieved of all anxiety, financially, with regard to the cost of the new wing now in progress and its equipment. Rapid progress has been made with the new wing, which it is hoped will be completed and formally opened early in the coming year.

### AN ASYLUM CONTRACT.

The contract for the building of a new wing to the Nottingham city asylum at Mapperley has been let to a local firm, and work is to be commenced in the first week of the New Year. The cost will be nearly £10,000.

### A CHILDREN'S INFIRMARY.

The Metropolitan Asylums Board has resolved to erect additional buildings and make certain alterations at the Children's Infirmary, Carshalton, which is being used for the reception and treatment of debilitated children from the metropolitan union infirmaries. The Infirmary caters for a large section of the metropolitan children, of whom some 745 are at present under treatment. The number of children fulfilling the conditions has been more than sufficient to fill the vacant beds. The new buildings will include the provision of isolation wards, and additional staff accommodation for nurses, the number of whom will have to be largely increased.

### METROPOLITAN ASYLUMS BOARD.

At an ordinary meeting of the managers of the Metropolitan Asylums Board at the Embankment offices a long discussion took place upon a proposal to ask the Local Government Board to authorise the use of the Orchard Hospital buildings at Dartford for unimprovable imbeciles. The committee pointed out that the continued residence of unimprovable imbeciles at Darenth was hampering the working of the scheme under which Darenth was made primarily a training centre. An amendment that the Lower Gore Farm Hospital be used for the purpose was agreed to. The transfer of Dr. J. B. Byles from the post of medical superintendent of the Gore Farm Hospital to that of medical superintendent of the Brook Hospital, subject to the approval of the Local Government Board and until further orders, was agreed to.

### ROYAL VICTORIA INFIRMARY, NEWCASTLE.

Noisy scenes accompanied the discussion of the report from the House Committee at the quarterly Court of Governors of the Royal Victoria Infirmary, Newcastle, held on November 25. The report recommended alterations in Statute 3 of the Institution, by which individual benefactors of £20 or more at one time should be governors for life. Previously the word "individual" had been omitted, and its insertion was challenged on the grounds that it was unfair to workmen who paid £10 for one year's governorship, and that the appointment for life of any man was not beneficial. After much disturbance an amendment was eventually carried whereby the paragraph was to be referred back to the House Committee. The remaining recommendations were : That any society, association, or company contributing in one year the sum of £10 or upwards should be entitled for every £10 to appoint one of their members a governor for the succeeding year; that the House Committee shall have authority to elect any individual a governor for one year in return for any sum collected, if not less than £10; and that the honorary officers of the Institution elected by the Selection Committee should be governors so long as they continued in office. These were carried; but the final proposal, that the House Committee should have authority to elect any individual a governor for life in acknowledgment of special services rendered to the Institution was referred back to the Committee for alteration, so that the election might rest with the governors.

